# Phase I/II study of DHA–paclitaxel in combination with carboplatin in patients with advanced malignant solid tumours

**DOI:** 10.1038/sj.bjc.6602196

**Published:** 2004-10-19

**Authors:** M Harries, A O'Donnell, M Scurr, S Reade, C Cole, I Judson, A Greystoke, C Twelves, S Kaye

**Affiliations:** 1 The CRUK Department of Medical Oncology, Royal Marsden Hospital Institute of Cancer Research, Downs Road, Sutton, Surrey SM2 5PT, UK; 2 The Beatson Oncology Centre, Glasgow, UK

**Keywords:** carboplatin, DHA-paclitaxel, phase I, taxane

## Abstract

DHA–paclitaxel is a conjugate of paclitaxel and the fatty acid, docosahexaenoic acid. Preclinical studies have demonstrated increased activity, relative to paclitaxel, with the potential for an improved therapeutic ratio. We conducted a phase I study to determine the maximum tolerated doses of DHA–paclitaxel and carboplatin when administered in combination. Two cohorts of patients were treated: carboplatin AUC 5 with DHA–paclitaxel 660 mg m^−2^ and carboplatin AUC 5 with DHA–paclitaxel 880 mg m^−2^. Both drugs were given on day 1 every 21 days. A total of 15 patients were enrolled with a median age of 59 years (range 33–71). All patients had advanced cancer refractory to standard treatment, performance status 0–2 and were without major organ dysfunction. A total of 54 cycles of treatment were delivered. No dose-limiting toxicity (DLT) was seen in the first cohort of three patients. In an expanded second cohort, neutropenia was the main DLT, occurring in the first cycle of treatment in five of 12 patients: three of these patients and one additional patient also experienced dose-limiting grade 3 transient rises in liver transaminases. No alopecia was seen and one patient developed clinically significant neuropathy. One partial response was seen in a patient with advanced adenocarcinoma of the oesophago-gastric junction and 12 patients had stable disease with a median time to progression of 184 days (range 60–506 days). The recommended phase II dose in pretreated patients is Carboplatin AUC 5 and DHA–paclitaxel 660 mg m^−2^ given every 21 days. Further studies with Carboplatin AUC 5 and DHA-paclitaxel 880 mg m^−2^, given every 28 days, are warranted in chemo-naive patients.

Paclitaxel is an effective and widely used anticancer drug with activity, either alone or in combination, against a range of solid tumours including breast, lung, urothelial and ovarian malignancies (Rowinsky and Donehower, 1995). Toxicities associated with paclitaxel, and the other commonly used taxane docetaxel, include hypersensitivity reactions, alopecia and myelosuppression. Paclitaxel can also cause significant peripheral neuropathy. Consequently, there is much interest in the development of new taxanes or taxane analogues with improved efficacy and less toxicity.

One approach to improve upon the therapeutic index of chemotherapy drugs is conjugation to fatty acids. In preclinical models, data indicate preferential uptake of such conjugates into tumours compared to normal tissue (Bradley *et al*, 2001b). Once in the cell, normal metabolism cleaves the fatty acid moiety to yield the active drug. Conjugation with a fatty acid would be expected to change the pharmacokinetic profile of the drug, possibly allowing for prolonged exposure of tumour cells to the chemotherapeutic agent and a reduction in the peak drug concentration, which might alter the toxicity profile.

DHA–paclitaxel is a covalent conjugate of paclitaxel and the essential fatty acid, docosahexaenoic acid (DHA) (Figure 1

**Figure 1:**
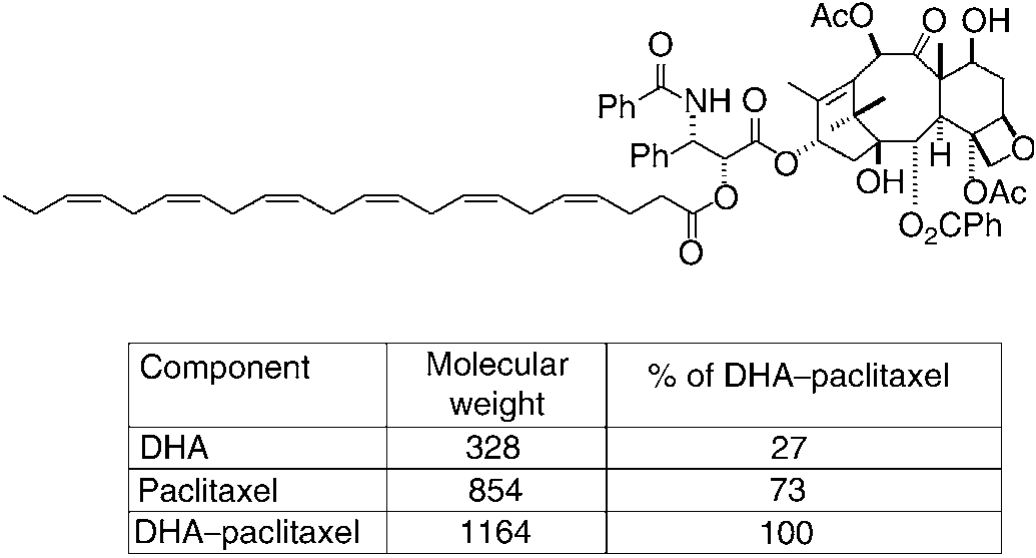
Molecular structure of DHA–paclitaxel.

). Docosahexaenoic acid is an omega-3, natural fatty acid, and a normal component of human breast milk. Bradley and co-workers have reported the preclinical activity, animal toxicity and pharmacokinetics of DHA–paclitaxel as follows.

The conjugate itself was found to have little *in vitro* cytotoxic activity. DHA–paclitaxel was found to be approximately 1000 times less active than paclitaxel against a panel of tumour cell lines. In contrast, studies in human xenograft tumour models demonstrated superior efficacy for DHA–paclitaxel compared with paclitaxel at equitoxic and equimolar doses. In a CD2F1 mouse/M109 tumour model, DHA–paclitaxel caused complete regression of 10 out of 10 tumours at its optimum dose (OD) of 120 mg kg^−1^ i.v. for 5 days. In contrast, no tumours were eliminated when paclitaxel was given at its OD of 20 mg kg^−1^ (zero out of 10), although slowing of tumour growth was seen. Similarly, in an HT-29 human colon cancer model, three partial responses and two complete responses were seen in five animals treated with DHA–paclitaxel. In this experiment, paclitaxel induced only temporary growth arrest (Bradley *et al*, 2001b).

The toxicity profile of DHA–paclitaxel was found to be superior to that of paclitaxel in mouse, dog and rat models. Bradley *et al* reported the maximum tolerated dose ratio of DHA–paclitaxel to paclitaxel, based on taxane molarity, to be 4.4 for mice, 3.6 for rats and 2.9 for dogs. No new toxicities were found for DHA–paclitaxel compared to paclitaxel in these studies. In a mouse model, hind limb paralysis seen with paclitaxel at its OD was not seen with DHA-paclitaxel (Bradley *et al*, 2001b).

Bradley and co-workers have also reported the tumour, plasma and muscle pharmacokinetics of DHA–paclitaxel in the M109 tumour-bearing mice. The AUC of DHA–paclitaxel in tumours was found to be much higher than that of paclitaxel, and the time during which tumour paclitaxel levels remained above a minimal therapeutic level was much longer (240 h compared to 16 h) (Bradley *et al*, 2001a).

Wolff and co-workers have reported the results of the first human phase I study conducted at the Johns Hopkins Hospital. A total of 24 patients were treated with DHA–paclitaxel over five dose levels (200–1100 mg m^−2^). The drug was found to be well tolerated with predictable toxicity and the dose-limiting toxicity (DLT) of myelosuppression. No patient developed alopecia or worse than grade 1 peripheral neuropathy. The pharmacokinetic data mirrored that seen in animal studies; the half-life of DHA–paclitaxel was found to be about seven times longer, the volume of distribution 100 times smaller and the clearance 300 times slower than paclitaxel. From this study the recommended dose for phase II evaluation of the single agent was determined to be 1100 mg m^−2^ (Wolff *et al*, 2003).

Combinations of a taxane and a platinum-containing compound are widely used in the treatment of many cancers (Harries and Gore, 2002; Hussain and James, 2003; Montagut *et al*, 2003; Ramalingam and Belani, 2002). The combination of DHA–paclitaxel and carboplatin has the potential to be a regimen with superior efficacy and less toxicity compared to currently used combinations such as paclitaxel–carboplatin or docetaxel–carboplatin. In this phase I trial, we aimed to define the maximum tolerated doses of the drugs when used in combination and to characterise the toxicities of a DHA–paclitaxel and carboplatin regimen.

## PATIENTS AND METHODS

### Patients

Patients were eligible for study entry if they fulfilled the following criteria: a histological diagnosis of an advanced solid tumour; had received conventional treatment for the disease; platinum-based therapy was considered appropriate; at least 4 weeks and recovery from previous antitumour therapy (6 weeks for Mitomycin-C and nitrosoureas); age>18 years; performance status 0, 1 or 2; neutrophil count ⩾1.5 × 10^9^ l; platelet count ⩾100 × 10^9^ l; adequate liver function (total bilirubin levels no greater than 1.5 times the normal limits and transaminases no higher than 2.5 times the normal limits); adequate renal function (glomerular filtration rate (GFR)>60 ml min^−1^, as measured using 51Cr-EDTA) and ability to give informed consent.

Exclusion criteria included prior therapy with any taxane; a past or current history of other cancer (except for curatively treated nonmelanoma skin cancer or carcinoma *in situ* of the cervix or other cancers if no current evidence of active disease was present); known or clinical evidence of central nervous system (CNS) metastases; peripheral neuropathy (of any aetiology, which was greater than grade 1); an unstable or serious concurrent medical condition.

The study was approved by the local hospital ethics committees.

### Study objectives

The primary objectives of the study were to assess the safety and tolerability of DHA–paclitaxel and carboplatin when administered in combination every 21 days, and to determine the maximum tolerated doses of the combination in this patient population.

### Treatment

Treatment was with 660 mg m^−2^ (starting dose) or 880 mg m^−2^ (second cohort) of DHA–paclitaxel administered intravenously over a 2-hour period. At 20–30 min following completion of DHA–paclitaxel infusion, carboplatin was administered intravenously over a period of 30 min. Carboplatin was given at a dose of AUC 5 (this dose was chosen as it is the commonly used dose of carboplatin when given with paclitaxel) with glomerular filtration rate determined by ^51^Cr-EDTA clearance. Treatment was administered on day 1 and was followed by 20 days of observation. Subsequent courses proceeded provided neutrophils were >1.5 × 10^9^ l^−1^ and platelets >100 × 10^9^ l^−1^ on the day of treatment. If on that day neutrophils and/or platelets had not reached the above thresholds, then treatment was deferred until recovery. Subsequent doses of DHA–paclitaxel were given with a 25% dose reduction. Likewise, if neutropenia associated with fever and/or sepsis, or thrombocytopenia associated with bleeding had occurred, then subsequent courses were given with a 25% dose reduction in the DHA–paclitaxel.

If a DLT resulted from a nonhaematological toxicity, the next DHA–paclitaxel dose treatment was reduced by one dose level. If any nonhaematological toxicity of grade 2 or 3 (except alopecia, and/or nausea, vomiting in patients who had not received optimal treatment with antiemetics) was present on the day of scheduled treatment, DHA–paclitaxel and carboplatin were withheld until the adverse event was resolved to grade 1 or baseline level. If any nonhaematological toxicity of grade 4 was present on the day of scheduled therapy, the patient was to withdraw from further participation in the study.

Treatment could continue for up to six courses or until evidence of disease progression, intolerable adverse events, patient refusal or investigator discretion. Tumour response was assessed every two courses using the Response Evaluation Criteria in Solid Tumors (RECIST). Safety parameters evaluated included adverse events and laboratory evaluations including haematology, serum biochemistry and urinalysis. Adverse events were graded according to the NCI Common Toxicity Criteria, Version 2.0.

### Dose escalation (Table 1)

**Table 1:** Dose escalation and dose limiting toxicity (DLT)

**Dose level**	***N***	**DHA-paclitaxel (mg m^−2^)**	**Carboplatin AUC**	**Number of patients with DLT**
1	3	660	5	0 of 3
2	12	880	5	6 of 12

The starting dose was DHA–paclitaxel 660 mg m^−2^ and carboplatin AUC 5. Initially, three patients were treated at this dose level, with the first patient being monitored for at least 2 weeks before additional patients were treated. Escalation to a new level was permitted when at least two of three patients had been evaluated for 3 weeks.

Dose escalation was according to the standard phase I criteria. Namely, if any patient experienced DLT that was considered to be related to the therapy of DHA–paclitaxel and carboplatin (with the exception of alopecia, and/or nausea or vomiting in patients who had not received optimal antiemetic therapy), three additional patients were enrolled at that dose level. Dose escalation then continued with a total of six patients enrolled at each dose until the recommended phase II dose level was reached. In the absence of toxicity that would define the recommended phase II dose level, drug dose levels were planned to be escalated as follows: Dose Level 1 – carboplatin AUC 5, DHA–paclitaxel 660 mg m^−2^; Dose Level 2 – carboplatin AUC 5, DHA–paclitaxel 880 mg m^−2^; Dose Level 3 – carboplatin AUC 5, DHA–paclitaxel 1100 mg m^−2^ (not reached).

Dose-limiting toxicity was defined as any one of the following adverse events: (1) grade 4 hematological toxicity: grade 4 neutropenia lasting at least 7 days or neutropenia complicated by fever or infection regardless of duration, thrombocytopenia <25 × 10^9^ l^−1^; (2) grade 3 or greater nonhaematological toxicities including diarrhoea (except alopecia, and/or nausea, vomiting in patients who had not received optimal treatment with antiemetics); (3) grade 2 haemorrhage or neurological (cerebellar) toxicities and (4) failure to recover from any drug-related toxicity by day 22.

It was planned that additional patients could be entered at the dose level at which escalation ceased, in order to better define the safety profile for phase II studies. The recommended phase II dose for the combination regimen was to be defined as the dose level at which dose escalation for each compound was to cease, or a lower dose, at the discretion of the Investigators.

## RESULTS

### Patients (Table 2)

**Table 2:** Patient characteristics

**Characteristic**	***N***
Entered/evaluable	15/15
Male/female	10/5
Median age (range)	59 (33–71)
*ECOG*^a^ *performance status*	
0	1
1	13
2	1
*Primary tumour site*	
Lung	5
Mesothelioma	5
Stomach	1
Oesophagogastric junction	1
Prostate	1
Unknown primary	1
Cervix	1
*Previous chemotherapy regimens*	
0	0
1	11
2 or more	4
Prior platinum	14
Previous radiotherapy	11
Other treatment (vaccines, etc.)	2

a Eastern Cancer Oncology Group.

Patient demographics are shown in Table 2. In total, 10 male and five female patients entered the study (total 15) with a median age of 59 years (range 33–71) and most (13 out of 15) with a baseline ECOG performance status of 1. The commonest cancer diagnoses were lung cancer and mesothelioma (five patients each), reflecting the nature of the referral pattern to the phase 1 unit in our institution. All patients had received prior chemotherapy and all but one had received prior platinum-containing chemotherapy. A total of 11 patients (73.3%) had received radiotherapy. All patients had progressed on or following previous treatments and no further conventional chemotherapy regimens were felt to be appropriate.

The first patient was enrolled on 2 February 2001 and the last patient came off study on 15 May 2002. All patients were evaluable for safety and efficacy. Five (33.3%) patients went off-study for disease progression. Four of the five patients had objective measurement of progressive disease and one patient had symptomatic deterioration due to disease. Five (33.3%) patients were removed from the study due to unacceptable toxicity, four patients (26.7%) refused further treatment and one (6.7%) was removed by decision of the investigators.

### Drug administration, dose adjustments and dose delays

The three patients in cohort 1 received all treatment without dose reduction or dose delay. Of the 12 patients in the second cohort, nine required a dose reduction of DHA–paclitaxel to 660 mg m^−2^. Six patients had the dose reduction after the first cycle. Five of the 12 patients (42%) had a delay of 1 week before delivery of the second cycle due to failure of their blood count to recover by day 22. Three patients (25%) in the second cohort received all their treatment at the planned dose of 880 mg m^−2^.

In total, 54 cycles of chemotherapy were delivered; 26 at a DHA–paclitaxel dose of 660 mg m^−2^ and 28 at a dose of 880 mg m^−2^. At the DHA–paclitaxel dose of 880 mg m^−2^, a 1-week dose delay for recovery of blood count was required in 30% of cycles. In all, 14% of the cycles delivered at the DHA–paclitaxel dose of 660 mg m^−2^ were delayed by 1 week (Table 3

**Table 3:** Summary of drug administration and dose adjustments

**DHA–paclitaxel dose level (mg m^−2^)**	**Number of patients**
660	3
880 → 660	9
880	3
Total	15
	
**DHA–paclitaxel dose level (mg m^−2^)**	**Number of courses given at dose level**
660	26
880	28
Total	54

Note: → indicates DHA–paclitaxel dose reduction.

).

### Toxicity

All 15 patients were evaluable for toxicity.

### Dose-limiting toxicity (Table 1)

No DLT occurred in the first cohort of three patients initially treated at the DHA–paclitaxel dose level of 660 mg m^−2^ and carboplatin AUC 5.

Two of the first six patients of the second cohort (DHA–paclitaxel 880 mg m^−2^ and carboplatin AUC 5) experienced haematological DLTs in treatment course 1: grade 4 neutropenia accompanied by fever; grade 3 thrombocytopenia and grade 4 neutropenia lasting 8 days. A third patient experienced the DLT of grade 3 increased transaminases (transient and asymptomatic).

At this point dose escalation ceased, but it was decided to obtain additional safety and toxicity data and to better define a recommended phase II dose by recruiting a further six patients at this dose level. Three of these six patients experienced DLTs in course 1: grade 4 neutropenia greater than 7 days and grade 3 increased transaminases; grade 4 neutropenia lasting 12 days and grade 3 increased transaminases; grade 4 neutropenia lasting eight days and grade 3 increased transaminases.

### Haematological toxicity

The haematological toxicities for the first course of carboplatin and DHA–paclitaxel are listed in Table 4

**Table 4:** Worst grade haematological toxicity first course

		**Grade**				
**Dose level DHA–paclitaxel**	**No of Pts. treated**	**0**	**1**	**2**	**3**	**4**
*Neutropenia*						
1 660	3	3	0	0	0	0
2 880	12	0	0	1	3	8
						
*Thrombocytopenia*						
1 660	3	3	0	0	0	0
2 880	12	10	1	0	0	0

Pts=patients.

. None of the three patients in the first cohort developed significant myelosuppression. Of the 12 patients in the second cohort treated with carboplatin AUC 5 and DHA–paclitaxel 880 mg m^−2^, 11 developed grade 3 or 4 neutropenia. For five patients, this was a DLT: four patients with grade 4 neutropenia lasting for more than 7 days and one further patient who developed neutropenic sepsis. One patient developed grade 3 thrombocytopenia.

When evaluating all cycles of chemotherapy, in total 10 patients experienced grade 4 neutropenia and three had an episode of neutropenic sepsis (two at grade 4 neutropenia and one at grade 3).

### Nonhaematological toxicity

Of the second cohort of patients, dose-limiting nonhaematological toxicity occurred in four patients in the first cycle of treatment, three of whom also experienced dose-limiting haematological toxicity. In all four cases, this was a short-lived asymptomatic grade 3 rise of liver transaminases. This was not felt to be clinically significant.

In subsequent courses, the worst nonhaematological toxicities per patient are recorded in Table 5

**Table 5:** Nonhaematological toxicity worst course per patient

	**Grade**				
**Toxicity**	**0**	**1**	**2**	**3**	**4**
Nausea, vomiting	6	4	4	1	0
Fatigue, weakness	3	0	8	4	0
Hypersensitivity	14	1	0	0	0
Transaminases	10	0	0	5	0
Neuropathy	7	5	2	1	0
Alopecia	14	1	0	0	0
Skin	13	2	0	0	0

. Grade 3 fatigue was seen in four patients. Grade 3 peripheral neuropathy occurred in one patient, grade 2 in two patients and grade 1 neuropathy in a further five.

Minimal alopecia was seen, with one patient developing grade 1 hair loss.

### Tumour response

One patient had a partial response, 12 patients had stable disease and two patients had progressive disease. The median time to progression for the patients with stable disease from day 1 of the first cycle of chemotherapy was 174 days (range 60–506 days).

The partial response occurred in a 59-year old man with advanced moderately differentiated carcinoma of the oesophago-gastric junction. Previously he had received two lines of chemotherapy with cisplatin/5-flurouracil and ECF (epirubicin, cisplatin and infusional 5-flurouracil). He had also received prior radiotherapy. The patient completed six courses of study therapy at a dose level of carboplatin AUC 5, DHA–paclitaxel 880 mg m^−2^. A partial response was first documented by CT scan during course 2, and was confirmed after course 4 (6 weeks after first documentation) and after course 6 (9 weeks after first documentation). During course 4, the patient experienced grade 3 paraesthesia probably related to study medication. At the time of the second confirmation of partial response (day 22 of course 6), the paraesthesia was unresolved, and the patient withdrew from the study due to this unacceptable toxicity. He subsequently developed progressive disease at 142 days from the beginning of chemotherapy.

## DISCUSSION

In this study, we have demonstrated that the combination of carboplatin and DHA–paclitaxel can be given to heavily pretreated patients with advanced cancer with the DLTs of neutropenia and transient transaminitis. Neutropenia appeared to be dose-related with none of the three patients in the first cohort but five of the 12 patients treated at the dose level DHA–paclitaxel 880 mg m^−2^ and carboplatin AUC 5 experiencing the DLT of neutropenia with the first cycle. In one patient, this was an episode of neutropenic sepsis and in the other four this was neutropenia lasting greater than 7 days (7, 8, 8 and 12 days). Neutropenia was the also the DLT seen in the single-agent phase I study (Wolff *et al*, 2003).

In contrast, only one of the 12 patients in the second cohort developed grade 3 thrombocytopenia. This suggests a possible platelet-sparing effect of the DHA–paclitaxel–carboplatin combination as is seen with carboplatin–paclitaxel (Guminski *et al*, 2001; Pertusini *et al*, 2001). The transaminitis seen in this study was short lived and asymptomatic. It is felt that this is unlikely to be of clinical significance.

Grade 3 peripheral neuropathy was seen in one patient after four courses of treatment in the same patient who had a partial response to treatment. Further studies will need to monitor peripheral neuropathy and in particular to determine if it is a feature of prolonged treatment with DHA–paclitaxel as can be seen with paclitaxel.

Fatigue was seen in several patients and is a feature of prolonged chemotherapy with taxane-containing regimens. Given the small sample size it is not possible to comment on whether the degree of fatigue seen in this study is above that one would expect for standard carboplatin/paclitaxel in this population.

The lack of alopecia is of great interest. The carboplatin–paclitaxel combination is standard treatment for women with advanced ovarian cancer. Hair loss in these women is often a major cause for concern and may lead some women to refuse treatment with taxanes. A regimen with equivalent toxicity but no alopecia would have significant advantages for many patients.

Although too few patients were treated on this study to comment on efficacy, the one partial response and 10 patients with stable disease was encouraging. The 6 month median time to progression seen in the patients with stable disease was also of interest. Further phase II studies of the combination in specific tumour types are required to more accurately estimate the true response rate.

We conclude that the recommended dose for further study in pretreated patients should be DHA–paclitaxel 660 mg m^−2^ and carboplatin AUC5 given on a 3-weekly basis. In addition, we believe this combination to be of sufficient interest to warrant testing in previously untreated patients, for example with non-small lung cancer. In such a chemotherapy-naïve population, consideration should be given to testing a 4-weekly regimen of DHA–paclitaxel 880 mg m^−2^ and carboplatin AUC 5. However, as such patients were not included in this study, such a trial would require a dose-validating phase with careful monitoring of haematological toxicity.
